# The Nexus of Aging and Substance Use: A Scoping Review of Therapeutic Modalities for Geriatric Substance Use Disorders

**DOI:** 10.7759/cureus.70313

**Published:** 2024-09-27

**Authors:** Noah Leton

**Affiliations:** 1 Physiology, Neuroscience and Behavioural Sciences, St. George's University, St. George's, GRD

**Keywords:** geriatric substance use disorders, interventions for substance misuse in older adults, management of substance use disorders in the elderly, substance misuse in older adults, substance use disorders in older adults

## Abstract

The insidious grip of substance use disorders (SUDs) manifests as a ubiquitous public health crisis, indiscriminately affecting individuals across the spectrum of age, gender, and socioeconomic status. While advancements in treatment offer a glimmer of hope, millions continue to grapple with the debilitating physical, psychological, and social consequences of addiction, particularly those involving alcohol and opioids. This crisis is further exacerbated by the alarming rise of SUDs among older adults. As the global population undergoes a process of demographic senescence, the escalating prevalence of SUDs in this demographic underscores the urgent need for nuanced interventions. This review explores the therapeutic landscape for managing SUDs in older adults, evaluating pharmacological and non-pharmacological treatment modalities. A detailed literature search was conducted using databases like PubMed, Google Scholar, and Scopus, and studies were selected based on their relevance to therapeutic interventions for older adults with SUDs, encompassing pharmacological and non-pharmacological modalities. The synthesized results provide an extensive overview of contemporary therapeutic approaches. The findings indicate that pharmacological interventions demonstrate varied effectiveness in managing opioid and alcohol use disorders, with each drug offering distinct benefits and limitations regarding safety, tolerability, and patient adherence. Non-pharmacological interventions provide critical psychological and social support, often requiring adaptations to meet elderly patients' needs effectively. Integrated care models, which combine pharmacological and non-pharmacological treatments, emerge as the most effective approach, addressing the comprehensive needs of elderly patients by leveraging multidisciplinary teams, centralized service access, and coordinated, patient-centered care. Implementing these models, however, requires overcoming significant resource and coordination challenges. Indeed, the confluence of a burgeoning geriatric population and escalating rates of SUDs necessitates the development and implementation of granular and integrated care protocols specifically designed for older adults. By employing such a targeted approach, optimism can be cultivated and the quality of life enhanced for this vulnerable and often overlooked segment of society. This ensures the fight against addiction extends its reach, leaving no one behind.

## Introduction and background

Substance use disorders (SUDs) represent a widespread public health crisis, impacting people from all walks of life without regard to demographic boundaries. While advancements in understanding and treating SUDs offer hope, the reality remains grim. Millions worldwide continue to battle addiction's debilitating grip, enduring profound physical, psychological, and social consequences [1]. The toll on society is immense, with soaring rates of morbidity and mortality linked to alcohol and opioid misuse [1]. This pervasive issue demands a multi-pronged attack - comprehensive interventions and unwavering commitment to treatment.

Within the wider discourse on SUDs, the aging population is a particularly vulnerable group that frequently goes unnoticed [2]. As the global population ages at an unprecedented pace, the stark reality is that the incidence of SUDs among older adults is on the rise. This demographic confronts unique challenges distinct from their younger counterparts [2]. Comorbid health conditions, polypharmacy (taking multiple medications), and age-related physiological changes create a complex web that hinders diagnosis and treatment. Traditional therapeutic approaches, often designed for a younger population, may not adequately address the multifaceted nature of SUDs in seniors [2].

This literature review investigates the complexities surrounding SUDs in the elderly, focusing on the effectiveness of targeted therapeutic interventions. Through critical evaluation of existing research and emerging interventions, this review aims to provide invaluable insights for clinicians and healthcare providers. This work aspires to advance the quality of treatment for seniors with SUDs, helping to guide them toward successful recovery.

Overview of substance use disorders

SUDs encompass a spectrum of conditions characterized by an uncontrollable urge to use psychoactive substances - alcohol, prescription medications, or illicit drugs - despite negative consequences. The compulsive nature of SUDs stems from neurobiological shifts in the brain's reward pathways, leading to a loss of control, persistent cravings, and the prioritization of substance use over other important aspects of life [3,4].

The global impact of SUDs is vast, affecting individuals from every background. While alcohol and opioids continue to dominate as the most commonly abused substances, the variety of misused substances is extensive, each with differing levels of harm and addiction risks. Demographic trends reveal that young adults and males are generally more prone to higher rates of substance use and associated challenges [2-4].

Regarding the classification and diagnosis of SUDs, the Diagnostic and Statistical Manual of Mental Disorders, Fifth Edition (DSM-5) outlines the criteria: (1) Taking the substance in larger amounts or over a longer period than intended. (2) Persistent desire or unsuccessful efforts to cut down or control use. (3) Spending a great deal of time obtaining, using, or recovering from the substance. (4) Craving or strong desire to use substance. (5) Recurrent use resulting in failure to fulfill major role obligations. (6) Continued use despite having persistent social and interpersonal problems. (7) Giving up or reducing important social, occupational, or recreational activities. (8) Recurrent use in physically hazardous situations. (9) Continued use despite knowledge of having a persistent or recurrent physical or psychological problem. (10) Tolerance, as defined by needing increased amounts of the substance to achieve the desired effect. (11) Withdrawal symptoms, which are specific to each substance. These features must be present for at least two months over the past one year. Furthermore, the disorder can be mild, moderate, or severe depending on the number of diagnostic criteria the individual meets (mild: two to three, moderate: four to five, severe: six or more). There are substance-specific categories like alcohol use disorder, opioid use disorder, etc., or polysubstance use, where the individual meets the criteria for two or more substances simultaneously [5].

An interrelation of various risk factors influences the onset of SUDs: genetic predisposition, neurobiological factors, psychological factors, socioeconomic status, other environmental factors, and sociocultural factors [5].

Challenges of substance use disorders in older adults

Firstly, the aging process brings about a variety of physiological changes that heighten both the risk and severity of SUDs [6-8]. Slower metabolism, altered body composition, and interactions with medications for chronic health conditions associated with aging can worsen existing health and create a cycle of decline [2,6-8].

Additionally, psychological and social factors significantly contribute to SUD development among seniors [4]. Retirement-related isolation, bereavement, and emotional distress can trigger substance use as a coping mechanism. Social isolation, exacerbated by societal stigma, further complicates the issue, hindering access to support and treatment [9,10].

Furthermore, older adults face significant barriers to SUD treatment, including limited mobility, transportation difficulties, financial constraints, and lack of age-appropriate programs [11,12]. The presence of cognitive decline and co-occurring health issues further complicate care [11,12].

Finally, addressing the surge in SUDs among older adults requires a significant shift in addiction treatment methodologies. Age-specific treatment programs, integrated care, telehealth, and cognitive-friendly interventions are needed to improve treatment outcomes [4,13,14].

Acknowledging the distinct susceptibilities and complex issues confronting older adults struggling with SUDs paves the way for the development of targeted interventions and the closure of the treatment access disparity [4,6-14]. This multifaceted strategy is crucial to fostering a more positive outlook for the aging population and curtailing the potential surge of geriatric SUDs.

A multifaceted threat to well-being

The notion that SUDs are predominantly a concern for younger populations is undergoing significant scrutiny. Emerging data indicates a troubling increase in SUD prevalence among older adults, presenting a formidable public health issue. This trend underscores the necessity for a comprehensive understanding of how SUDs affect seniors, impacting their physical health, mental well-being, and social interactions [2,4].

SUDs in seniors interfere with daily activities, leading to greater reliance on caregivers and fostering feelings of helplessness [2,9,15]. The associated stigma often drives social withdrawal, as many avoid interactions due to fear or shame, further intensifying loneliness and reinforcing substance use as a coping strategy [2,9,15].

Furthermore, seniors with SUDs experience intense financial strain, as the combined costs of substances, treatment, and health complications can overwhelm their fixed incomes [16]. SUDs also exacerbate chronic diseases like diabetes and heart conditions, complicating treatment and straining healthcare resources [16,17]. Additionally, substance use impairs cognitive and motor functions, increasing the risk of falls and injuries, which necessitates greater dependence on long-term care [16,17].

Substance abuse in seniors also compromises their immune defenses, increasing susceptibility to infections like pneumonia and UTIs, which results in extended hospital stays and slower recovery [18-21]. Additionally, substance use frequently diminishes appetite and self-care, leading to malnutrition and further weakening their already fragile health [19,20].

In the same vein, SUDs in seniors accelerate cognitive decline, worsening conditions like dementia and Alzheimer's, leading to memory lapses, impaired judgment, and increased dependence [18,22]. These disorders also heighten emotional issues such as depression, anxiety, and hopelessness, creating a perfect storm for a rapid decline in overall well-being [20].

Finally, in this demographic, SUDs significantly heighten the risk of suicide [23,24]. The combination of physical and mental health struggles, coupled with social isolation, creates a dangerous scenario for self-harm [24]. The lack of adequate mental health care and support further deepens their despair, making early intervention critical to preventing such tragedies and restoring hope for this at-risk group [23,24].

Public health and economic implications

Substance use among the elderly is an increasing public health issue, worsening chronic illnesses and raising the risk of infections, accidents, and overdoses [19,25]. This surge contributes to more hospitalizations, emergency visits, and long-term care requirements, further straining already overburdened healthcare systems [19,25]. Managing SUDs in seniors demands specialized programs and continuous care, but the growing need for geriatric and addiction services leads to service delays and gaps, complicating treatment [4,12].

Seniors with SUDs are also at a greater risk of infectious diseases like hepatitis and HIV, as well as infective endocarditis, driven by associated risky behaviors, necessitating focused public health interventions [26-28]. The interaction between SUDs and mental health disorders, such as depression and anxiety, exacerbates both conditions, creating a higher demand for specialized mental health services and straining existing resources [29,30].

The economic burden is significant, with direct costs arising from increased spending on treatment, hospital stays, and medications, and indirect costs from lost productivity as family members take on caregiving roles [31-33]. The expenses of long-term care also escalate, particularly given the complex needs of older adults with SUDs [32].

The rising demand for social services like housing and in-home care adds further pressure to social service agencies, forcing tough resource allocation decisions in a climate of limited funding [34,35]. The economic impact extends to policymakers and insurers, as escalating healthcare costs related to SUDs threaten the viability of both public and private insurance systems, resulting in higher premiums and increased pressure on Medicare and Medicaid [17,36,37].

Management approaches

Effectively managing these disorders requires a comprehensive strategy that addresses the unique needs of this population. A thorough assessment is the cornerstone of effective treatment. This should be a holistic evaluation, delving into the medical, psychological, and social aspects contributing to substance use in seniors​ [38]. This in-depth evaluation can be further enhanced by utilizing screening tools specifically adapted for older adults, such as the Geriatric Depression Scale (GDS) and the Alcohol Use Disorders Identification Test (AUDIT). These tools allow healthcare professionals to make accurate diagnoses of SUDs, paving the way for successful treatment plans [39,40].

Treatment programs must be adaptable to address the distinct challenges faced by seniors with SUDs effectively [38]. This may involve modifying medication dosages in response to age-related physiological alterations, employing less-intensive detoxification strategies to minimize discomfort, and providing additional support to manage any physical limitations that may arise [38,41]. Incorporating geriatric care principles into the treatment plan is essential. This includes addressing chronic pain management, offering support for potential cognitive decline, and providing assistance with mobility limitations commonly experienced by this population [42].

While medication and basic medical care play a role, behavioral therapies offer a crucial, individualized approach to SUD treatment for seniors [38,43,44]. Cognitive-behavioral therapy (CBT) proves particularly valuable by equipping seniors with the tools to identify and modify their substance use patterns, even when accounting for any potential cognitive decline [43,44]. This tailored approach optimizes CBT's effectiveness for each senior. Motivational interviewing (MI) serves as a cornerstone, fostering a collaborative environment where seniors can discover their motivation for change and actively engage in the treatment process [43].

The efficacy of medication-assisted treatment (MAT) in alleviating withdrawal symptoms and curbing cravings is well-documented [38,45,46]. Pharmaceuticals like methadone, buprenorphine, and naltrexone play a crucial role, yet vigilant supervision is necessary to avert potential interactions with other medications commonly administered to the elderly [38,45,46]. Consistent follow-up visits facilitate dosage modifications, guaranteeing that the therapeutic regimen remains safe and efficacious [38].

The role of support services in recovery is indispensable [38,47]. Involvement of family, friends, and community networks provides seniors with emotional support and helps alleviate isolation. Peer support groups designed for older adults with SUDs offer significant peer support and reduce the stigma of seeking help [38,47]. For seniors with limited mobility or those in remote areas, home-based care options, including telehealth services, offer convenient and accessible support [47]. Regular home visits by healthcare professionals ensure compliance with treatment plans and monitor recovery progress [47].

Lifestyle modifications are an integral component of effective SUD management [38,48]. Advocating for healthy practices such as routine physical activity, optimal nutrition, and stress reduction techniques can significantly improve holistic health outcomes and support recovery processes [38,48,49]. Engaging seniors in structured social and recreational activities can establish a sense of purpose and community, thereby mitigating the potential for relapse [38,48].

Furthermore, with concomitant mental health disorders, such as depression and anxiety, posing a significant challenge to successful SUD treatment in seniors [38,50,51], integrating mental health services directly within SUD treatment programs ensures comprehensive management of these co-occurring conditions [50,51]. A spectrum of therapeutic options, including individual counseling, group therapy, and psychiatric support, should be readily available to address the unique mental health needs of each senior [50,51]. Incorporating mindfulness-based interventions and stress-reduction techniques like meditation, yoga, and relaxation exercises can foster emotional well-being and equip seniors with healthy coping mechanisms for managing emotional distress [52,53].

Finally, integral to a comprehensive SUD management approach, education and prevention programs serve to heighten awareness, foster critical life skills, mobilize community involvement, and ensure early intervention [38]. These efforts are instrumental in reducing both the incidence and the ramifications of substance use disorders [38].

Inadequate care due to gaps in research

Although SUDs are an increasing concern among seniors, substantial research gaps hinder effective interventions for this demographic: limited age-related data, lack of clarity on risk determinants, innovative technological approaches, effectiveness and adaptation in treatment protocols, preventive measures, and early intervention, and sociocultural factors.

By addressing these research gaps, a deeper understanding of this issue can be cultivated and healthcare professionals can be equipped with the tools they need to improve health outcomes and quality of life for this vulnerable and growing population.

Study rationale

For policymakers, healthcare providers, and researchers, this study offers valuable insights into SUDs among seniors. It explores the public health significance, the challenges specific to this population, the knowledge gaps that need to be addressed, and the potential economic and social impacts.

Aim of study

This study aims to examine the effectiveness of various therapeutic modalities in reducing substance use in older adults, improving physical health, enhancing mental well-being, and improving the overall quality of life. It also assesses the comparative efficacies of the various therapeutic modalities.

## Review

Extensive searches were conducted on PubMed, Google Scholar, and Scopus. Studies were selected based on their relevance to therapeutic interventions for older adults with SUDs, encompassing pharmacological and non-pharmacological modalities. Search terms such as 'geriatric substance use disorders,' 'substance use disorders in older adults', and 'interventions for substance use disorders in the elderly,' were used to identify relevant studies. The main organization of the review is based on the type of intervention, complemented by comparative analyses of various interventions.

Pharmacologic approaches

Clinically proven pharmacological interventions, often referred to as MAT, play a vital role in managing SUDs. Backed by robust research, these interventions demonstrably suppress cravings for the addictive substance, decrease the likelihood of relapse, and effectively support individuals in managing withdrawal symptoms [38,45,46]. This section delves into the application of MAT for opioid-use disorder (OUD), alcohol-use disorder (AUD), and nicotine-use disorder (NUD). Importantly, it's crucial to recognize the vast array of medications available to target a wider range of substances, as well as co-occurring mental health conditions like depression and anxiety (antidepressants and anxiolytics, respectively).

Buprenorphine

Studies, like that of Konakanchi and Sethi, demonstrate that the distinct pharmacological profile of buprenorphine makes it an optimal treatment for OUD in older adults [54]. Like the study by Konakanchi and Sethi, Dufort and Samaan reveal that it effectively reduces opioid use and cravings as well as withdrawal symptoms, enhancing its utility in this population [54,55]. One key advantage of buprenorphine is its extended half-life, which permits less frequent dosing compared to other opioid agonists [54,55]. This feature is particularly beneficial for older adults, who may struggle with adherence to daily medication regimens. By reducing the burden of strict dosing schedules, buprenorphine improves compliance and supports sustained recovery [54,55]. The safety profile of buprenorphine has also been demonstrated in this age group [55,56]. Unlike full opioid agonists, it carries a lower risk of respiratory depression [55]. This reduced risk translates into a significantly lower likelihood of fatal overdose, addressing a major public health issue associated with opioid misuse [56]. Additionally, Davis et al. revealed that buprenorphine is effective in the management of chronic pain in older adults [57]. This improves their physical comfort and enhances their overall quality of life, enabling them to engage more fully in daily activities. Dufort and Samaan also bring to the fore the accessibility to buprenorphine, further strengthening its suitability for older adults [55]. Collectively, these benefits underscore the role of buprenorphine as a well-tolerated and effective treatment for OUD in older adults, particularly those with complex medical needs [54,55]. Its safety, efficacy, and flexibility make it a critical tool in managing both OUD and chronic pain in this vulnerable population.

*Methadone* 

Methadone, a long-acting opioid agonist, is notably effective in managing OUD by reducing withdrawal symptoms and cravings without inducing euphoria. The medication's once-daily dosing maintains stable opioid levels, which is essential for preventing relapse. However, the Konakanchi and Sethi study shows its use requires careful monitoring by healthcare professionals due to potential side effects and drug interactions​ [54]. Like the Konakanchi and Sethi study, Leal and January revealed that in the elderly, the application of methadone for medication-assisted treatment (MOUD) is limited by preexisting conditions such as cardiac conduction abnormalities, which are more prevalent in this age group compared to the general population [54,58]. Specifically, methadone has been shown to prolong the QT interval, leading to severe arrhythmias, and can also cause respiratory depression and interact with various medications commonly prescribed to elderly patients​ [54]. The study by Konakanchi and Sethi also reveals the issues of accessibility to methadone by the elderly, as they have to report daily to the health facility [54]. Despite these risks, the effectiveness of methadone is well-supported by research. Although some studies show that the retention rates of methadone and buprenorphine are generally the same [59], a study by Lim et al. shows that methadone has the highest treatment retention rates for OUD (64.1%), significantly enhancing patient engagement in treatment compared to buprenorphine (54.3%) and naltrexone (41.0%) [60]. This engagement is critical for long-term recovery from OUD​ [60]. The study also highlighted the ranking of methadone as the highest when it comes to treatment based on Surface Under the Cumulative Ranking (0.901), surpassing both buprenorphine (0.559) and naltrexone (0.257) [60]. Moreover, methadone treatment offers financial benefits for elderly patients with OUD [61]. Research, like that of Kendall et al., indicates a decrease in hospital admissions and emergency department visits when on methadone (with an adjusted odds ratio - OR of 0.5), leading to lower healthcare costs [61]. The study by Tran et al. complements other studies as regards better management of their OUD and related health problems, resulting in an improved quality of life​ [62]. Overall, the positive outcomes associated with methadone treatment strongly support its continued use as a cornerstone therapy for OUD, especially for elderly patients, despite the need for careful monitoring​.

Naltrexone

Naltrexone emerges as a versatile medication in the treatment of SUDs in older adults. Acting as an opioid antagonist, it effectively combats OUD by curbing cravings and preventing relapse [63-65]. While its precise mechanism of action for AUD remains unclear, naltrexone has nonetheless demonstrated significant effectiveness in this realm as well [63-65]. For OUD, particularly, the extended-release formulation of naltrexone, XR-NTX, has garnered substantial support from research. Studies have consistently highlighted its efficacy in reducing cravings and relapse rates. One such study by Lee et al., although not exclusively carried out on older adults, demonstrated that participants on XR-NTX had an increased median relapse time compared to those on the non-extended form (10.5 vs. 5.0 weeks, P<0.001; hazard ratio, 0.49; 95% confidence interval - CI, 0.36 to 0.68) [64]. The study also reported a lower relapse rate (43% vs. 64% of participants, P<0.001; OR, 0.43; 95% CI, 0.28 to 0.65), and a higher rate of opioid-negative urine samples (74% vs. 56%, P<0.001; OR, 2.30; 95% CI, 1.48 to 3.54) [64]. Similarly, a study by Steffens et al. highlighted the effectiveness of this medication in the geriatric population [63]. The study underscored the higher adherence rates of older adults to treatment with naltrexone [63]. However, Lee et al. also revealed that XR-NTX resulted in more adverse effects than regular naltrexone [64]. In the treatment of patients with OUD and co-occurring anxiety and depression, a study by Latif et al. revealed that symptoms of both anxiety and depression decreased significantly [65]. Additionally, as demonstrated in the Mahmoud et al. study, the combination of MAT (including XR-NTX therapy) with telehealth has emerged as a potent means in overcoming access barriers often faced by individuals, including the elderly, with SUDs [66].

For AUD, naltrexone has also proven to be an essential resource. Research, like that of Murphy et al., demonstrates a significant decrease in the number of drinking days and heavy drinking days per month [67]. Results from this study showed that weighted mean difference was −2.0 [95% CI = −3.4, −0.6; P = 0.03] in favor of extended-release naltrexone for drinking days per month and −1.2 (95% CI = −0.2, −2.1; P = 0.02) for heavy drinking days per month, indicating that treatment resulted in two fewer drinking days per month and 1.2 fewer heavy drinking days per month compared with placebo [67].

In conclusion, research strongly supports the safety and efficacy of naltrexone as a treatment for both OUD and AUD in elderly populations. XR-NTX offers a particularly promising option due to its long-term effectiveness, improved medication adherence, and minimal side effects.

Disulfiram

In the management of AUD disulfiram employs a more indirect, yet potent, strategy: deterrence [68]. Disulfiram competitively inhibits aldehyde dehydrogenase, a crucial enzyme responsible for breaking down acetaldehyde, a byproduct of alcohol metabolism [68]. This inhibition leads to a buildup of acetaldehyde in the body after alcohol consumption which triggers a cascade of unpleasant physiological symptoms, creating a powerful negative reinforcement association with alcohol consumption [68]. Regarding the effectiveness of the medication, a study by Koob pointed out that associated adherence issues decreased the effectiveness, with close supervision increasing adherence and ultimately effectiveness [69]. The effectiveness of disulfiram was further underscored by Laaksonen et al., who showed that disulfiram was more effective than acamprosate or naltrexone in the reduction of heavy drinking and the average weekly alcohol intake [70]. The study, which consisted of both young and older adults, also demonstrated an increase in the time taken to consume the first drink and, the number of abstinent days [70]. In the same vein, the study demonstrated a synergistic effect between disulfiram and CBT in reducing alcohol consumption, extending periods of abstinence, as well as reducing symptoms of depression, and improving quality of life [70]. Disulfiram was also found to be well-tolerated, with the Mutschler et al. study reporting tiredness (which ceased within six weeks of treatment) in 100% of study participants [71]. The study also reported mild disulfiram-ethanol reaction after alcohol ingestion, and no serious adverse effects, including disulfiram hepatoxicity, were reported [71]. However, a study by Lin et al. revealed that since disulfiram is contraindicated in the presence of cerebrovascular disease, and peripheral neuropathy, amongst others, which are common in older adults, disulfiram should be avoided in this age group [4]. Regarding long-term effectiveness, supervised disulfiram use demonstrates a positive track record. The research by Mutschler et al., showed a cumulative alcohol abstinence of 16.88 ± 20.48 months [71]. To conclude on disulfiram, it presents an effective means for managing AUD, especially when administered with supervision. However, a significant limitation exists - the paucity of research on the effectiveness of disulfiram in older adults. While its potential benefits are undeniable, further studies are needed to determine the safety and efficacy of disulfiram in this vulnerable population.

Acamprosate

With chronic alcohol disrupting the delicate equilibrium of glutamatergic neurotransmission, acamprosate acts as a modulator, restoring this balance and facilitating the normalization of brain activity as recovering individuals rebuild their lives [72]. This targeted approach translates into demonstrably positive outcomes. Several studies have confirmed the effectiveness of acamprosate in promoting abstinence for patients with AUD. One such study was a study by Celik et al. [72]. This study revealed that acamprosate prolonged abstinence, although it had no significant effect on binge drinking [72]. The effectiveness of acamprosate increases when combined with psychotherapy, as revealed by the Plosker study [73]. The study demonstrated that combining acamprosate with psychotherapy for three to 12 months yielded various key outcomes, including a complete abstinence rate, an increase in alcohol-free days, and the median time taken to have the first drink [73]. Regarding the tolerability of acamprosate, the study by Plosker also revealed that the medication is well tolerated [73]. Side effects like anxiety, diarrhea, and headache have been reported [72]. The drug is also contraindicated in individuals with renal impairment [72,73]. While research specifically focused on older adults is limited, and further research is needed to definitively establish its effectiveness in this age group, acamprosate has shown promise in this population. Its tolerability, with minimal side effects, makes it a potentially valuable option for relapse prevention in elderly patients with AUD [74].

Nicotine Replacement Therapy

Nicotine replacement therapy (NRT) stands as a pivotal pharmacological intervention in the management of smoking addiction. It provides a meticulously controlled dose of nicotine, minus the multitude of harmful toxins found in cigarettes. It helps alleviate withdrawal symptoms and cravings, acting as a bridge for smokers attempting to quit. Studies have confirmed the effectiveness of NRT, with Stead et al. revealing that NRT increases the rate of quitting smoking by 50 - 70% [75]. This study also demonstrated that NRT caters to individual needs and preferences by offering various delivery methods, including transdermal patches, lozenges, and sprays [75]. This adaptability enhances patient adoption and adherence to the therapy. For elderly smokers, the study by Scholz et al. revealed that those over 60 years of age and using NRT alone had a significantly increased cessation success (OR 2.34, 95% CI: 1.36 to 4.04, p = 0.002) [76]. The combination of NRT and behavioral therapy is highly effective as well, especially in older patients, as demonstrated by Khan et al., who underscored the benefits of combining pharmacotherapy and behavioral therapy [77]. Safety and tolerability are crucial considerations, especially for older populations. Fortunately, research indicates that NRT is safe and generally well-tolerated, with patches being the easiest form to use, although inadequate for relief of acute cravings [75]. While some may experience mild side effects such as localized skin irritation or occasional gastrointestinal discomfort, these are typically manageable and do not significantly impact treatment adherence [75]. Chest pain and palpitations have been reported with the use of NRT, but these are rare occurring at a rate of 2.5% in the NRT groups compared with 1.4% in the control groups [75]. Long-term success hinges on consistent use and regular monitoring. Studies investigating long-term outcomes suggest that sustained NRT use, coupled with regular follow-up, is a successful strategy for maintaining smoking cessation [75]. To conclude, considering the unique challenges faced by elderly smokers, NRT is vital for achieving and sustaining smoking cessation. Its targeted approach, diverse delivery methods, and favorable safety profile make it a compelling option for this demographic.

Bupropion

Bupropion is an antidepressant medication that has also been licensed as a pharmacological intervention for smoking cessation. Unlike traditional antidepressants that primarily target serotonin reuptake, bupropion employs a "dual reuptake inhibition" mechanism, simultaneously increasing levels of norepinephrine and dopamine in the central nervous system [78]. This targeted approach not only addresses depressive symptoms that may co-occur with NUD but also effectively modulates neurotransmitter systems implicated in reward processing and craving reduction, ultimately aiding smoking cessation across various age groups, including the elderly population [78]. Clinical trials investigating the efficacy of bupropion for smoking cessation have yielded encouraging results. Approximately 20% of smokers successfully stop and remain non-smoking for one year with bupropion therapy [78]. It was also revealed that bupropion therapy doubled the odds of quitting smoking over placebo (OR 2.06, 95% CI 1.77 to 2.40) [78]. The medication can also be prescribed to patients with other disease conditions such as cardiovascular disease and chronic obstructive pulmonary disease, having similar success rates when compared to healthy smokers (27% bupropion vs 11% placebo at 26 weeks, 22% vs 9% at 52 weeks, p < 0.001) [78]. The sustained-release (SR) bupropion is more effective than placebo in increasing quit rates [79]. This positive effect is further bolstered by evidence of higher continuous abstinence rates at the critical six-month follow-up mark, highlighting the potential for sustained smoking cessation with bupropion treatment [78]. A study by Elhassan and Chow revealed that, even in elderly patients, SR bupropion is effective and doubles the rate of smoking cessation [80]. This study also demonstrated that advanced age was one of the factors for successful treatment with bupropion [80]. Regarding safety and tolerability, research suggests that bupropion exhibits a favorable safety profile and is well tolerated in this population, even in the presence of comorbidities [80]. While some patients may experience mild and transient side effects such as insomnia or xerostomia (dry mouth), these are manageable and do not significantly impact treatment adherence [80]. Furthermore, studies suggest that bupropion is a cost-effective option for treating smoking cessation, and superior to NRT in that regard, giving an incremental cost per quality-adjusted life year gained (QALY) for bupropion of approximately 30% less than that for NRT [79]. All in all, bupropion's dual-mechanism approach, safety profile, and potential for improving mental well-being make it an asset for the management of NUD in elderly populations. Further research is warranted to explore the long-term efficacy and optimal treatment protocols for bupropion use in this population.

*Varenicline * 

Varenicline acts as a partial agonist at the nicotinic acetylcholine receptor (nAChR), simultaneously reducing the intensity of cravings and withdrawal symptoms associated with NUD cessation, while concurrently blunting the rewarding effects of smoking [81]. Studies have yielded compelling evidence regarding the efficacy of varenicline in promoting smoking abstinence in geriatric populations. In a study by Chang et al., it was shown that not only were elderly smokers receiving varenicline more likely to achieve cessation than those using NRT (OR, 3.22; 95%CI, 1.23-8.43), but varenicline was also prescribed more than NRT (78.3% vs. 21.7%) in clinical settings [82]. However, a previous study by Chang et al., showed that varenicline did not offer greater effectiveness than NRT in older adults, with its effectiveness being more in smokers aged 54 years and younger [83]. The reasons underlying this disparity remain unclear and warrant further investigation. Potential contributing factors may include age-related variations in drug metabolism or the higher prevalence of comorbidities in geriatric populations, which could influence treatment response [83]. The positive impact of varenicline extends beyond the immediate cessation attempt, with research suggesting that the effectiveness of varenicline can persist for as long as 104 weeks, particularly when combined with non-pharmacological interventions such as behavioral counseling, than when only counseling is instituted (29.2% vs 18.8%) [84]. Regarding previous safety concerns with varenicline use in the elderly, particularly those related to potential neuropsychiatric and cardiovascular sequelae, these have been largely allayed [85]. However, a degree of caution is still recommended due to potential differences in drug tolerance levels between younger and older populations [83]. Concerning tolerability, there were no differences in side effects between the varenicline and placebo groups, with the most common side effects being gastrointestinal disturbances [80]. Advancements in treatment strategies involve "adaptive treatment approaches" utilizing varenicline as demonstrated by Davis et al. and the Rose and Behm studies [86,87]. These approaches involve initiating varenicline treatment earlier in the smoking cessation process and tailoring the dosage based on individual patient response, particularly in elderly smokers [87,88]. Studies suggest that this flexible approach can significantly boost cessation rates in this population [86,87]. In conclusion, varenicline is a viable pharmacological agent for smoking cessation in geriatric populations. Its targeted action on nAChRs, demonstrably long-term effectiveness, and potential for adaptive treatment approaches make it a compelling option for older adults seeking to quit smoking.

Comparative analysis of pharmacologic agents

Medication-Assisted Treatment for Opioid-Use Disorder

Choosing the most effective medication for OUD treatment in elderly patients requires an understanding of each option's advantages and disadvantages. Here, buprenorphine, methadone, and naltrexone are compared. Table 1 summarizes the comparative analysis.

**Table 1:** Comparative analysis of medication-assisted treatment for opioid-use disorder OUD: opioid-use disorder, AUD: alcohol-use disorder

Aspect	Buprenorphine	Methadone	Naltrexone
Mechanism of Action	Partial opioid agonist	Full opioid agonist	Opioid antagonist
Effectiveness in Reducing Cravings	Highly effective in reducing opioid use, cravings, and withdrawal symptoms in older adults [54,55].	Effective in reducing withdrawal symptoms and cravings without inducing euphoria [54]. Has the highest treatment retention rates (64.1%) compared to buprenorphine (54.3%) and naltrexone (41.0%) [60].	Effective in curbing cravings and preventing relapse for OUD. Extended-release formulation (XR-NTX) increases median relapse time and reduces relapse rates [64].
Safety and Tolerability	Lower risk of respiratory depression and overdose compared to full opioid agonists [55,56]. Well-tolerated in older adults with complex medical needs [54,55].	Risk of respiratory depression, QT interval prolongation leading to severe arrhythmias, and drug interactions [54,58]. Requires careful monitoring, particularly in older adults with preexisting conditions [54,58].	Safe with minimal side effects. However, extended-release naltrexone (XR-NTX) may cause more adverse effects than regular naltrexone [64]. Generally well-tolerated in older adults [63].
Accessibility	More accessible, reducing barriers to treatment [55].	Limited accessibility due to the need for daily visits to healthcare facilities, which can be a challenge for elderly patients [54].	Telehealth combined with XR-NTX improves access to treatment [66].
Impact on Treatment Engagement	Buprenorphine supports sustained recovery by improving compliance with less frequent dosing [54,55].	Methadone has the highest treatment retention rates and is ranked highest for treatment based on Surface Under the Cumulative Ranking [60].	Higher adherence rates in older adults, especially with extended-release formulation [63].
Impact on Co-morbidities	Safe and effective for older adults with complex medical needs [54,55].	Requires careful consideration of preexisting conditions, particularly cardiac issues [54,58].	Effective in reducing symptoms of anxiety and depression in patients with OUD and co-occurring mental health conditions [65].
Overall Suitability for Older Adults	Well-tolerated, effective, and flexible treatment option, particularly for those with complex medical needs [54,55].	Effective but requires careful monitoring due to potential side effects and drug interactions, limiting its use in elderly patients with preexisting conditions [54,58].	Safe, effective, and particularly promising in extended-release form (XR-NTX) for OUD and AUD in elderly populations. Improved adherence and minimal side effects make it a strong candidate for this population [63,64].

In conclusion, a thorough healthcare evaluation is essential to identify the most appropriate medication for each patient with OUD. Buprenorphine stands out for its safety, tolerability, and flexible dosing, making it a particularly suitable option for elderly patients with complex medical needs. Methadone, while highly effective and offering the best treatment retention rates, requires careful monitoring due to its associated risks, particularly in older adults with preexisting cardiac conditions. Naltrexone, especially in its extended-release form, offers a safe and effective alternative with minimal side effects and improved adherence, making it a promising choice for elderly patients with OUD and co-occurring mental health conditions. Ultimately, a personalized treatment plan tailored to the individual needs of elderly patients ensures the most effective management of OUD.

Medication-Assisted Treatment for Alcohol-Use Disorder

For elderly individuals with AUD, choosing the most effective and safe medication requires careful consideration of the available options: naltrexone, disulfiram, and acamprosate. Table 2 summarizes the comparative analysis.

**Table 2:** Comparative analysis of medication-assisted treatment for alcohol-use disorder OUD: opioid-use disorder, AUD: alcohol-use disorder

Aspect	Naltrexone		Disulfiram		Acamprosate
Mechanism of Action	Opioid antagonist also used for AUD, though the exact mechanism in AUD is less clear [63-65].		Inhibits aldehyde dehydrogenase, causing a buildup of acetaldehyde after alcohol consumption, leading to unpleasant physiological effects that deter alcohol use [68]		Modulates glutamatergic neurotransmission, restoring balance disrupted by chronic alcohol use, and facilitates normalization of brain activity [72].
Effectiveness in Reducing Cravings	Effective in reducing cravings and relapse rates in OUD, particularly with extended-release formulations (XR-NTX). Also effective in reducing drinking days and heavy drinking days in AUD [64,67].		Effectiveness is highly dependent on adherence. When supervised, disulfiram is effective in reducing heavy drinking, average weekly alcohol intake, and prolonging periods of abstinence [69,70].		Effective in promoting abstinence, especially when combined with psychotherapy. However, it is less effective in preventing binge drinking [72,73].
Safety and Tolerability	Generally well-tolerated, with minimal side effects. Extended-release naltrexone may cause more adverse effects than regular naltrexone. Serious side effects include potential liver damage and depression [64].		Well-tolerated when supervised. Common side effects include tiredness and mild disulfiram-ethanol reactions. Contraindicated in patients with cerebrovascular disease and peripheral neuropathy [4]. No serious adverse effects reported, though further research on its use in older adults is needed [71].		Well-tolerated with side effects such as anxiety, diarrhea, and headache. Contraindicated in individuals with renal impairment [72,73].
Accessibility	XR-NTX combined with telehealth services has improved accessibility, particularly for older adults and those in remote areas [66].		Accessibility may be limited by the need for daily dosing and supervision to ensure adherence, which can be challenging in older adults [68].		Accessibility is generally good, but the need for multiple daily doses may affect adherence, especially in older adults [72].
Impact on Treatment Engagement	High adherence rates with extended-release formulations, particularly in older adults. Improves engagement by reducing the frequency of dosing [63,64].		High engagement when supervised, but poor adherence without supervision can reduce effectiveness [69].		Effective in increasing alcohol-free days and promoting abstinence when combined with psychotherapy, which can enhance patient engagement [73].
Impact on Co-morbidities	Effective in reducing symptoms of anxiety and depression in patients with OUD and co-occurring mental health conditions [65].		Has been shown to reduce symptoms of depression when combined with Cognitive Behavioral Therapy (CBT), though more research is needed specifically in older adults [70].		Well-tolerated and potentially effective in older adults, though research specific to this population is limited. More studies are needed to understand its full impact on older adults with co-occurring conditions [74].
Overall Suitability for Older Adults	Safe and effective for OUD and AUD in elderly populations, particularly in extended-release form (XR-NTX). Offers a promising option due to its long-term effectiveness, improved adherence, and minimal side effects [63,64].		Effective, especially when used under supervision, but the need for daily adherence and the potential for unpleasant reactions may limit its suitability in older adults without close monitoring. Lack of research specifically focused on older adults is a concern [70,71].		Promising for promoting abstinence in older adults with AUD, particularly when combined with psychotherapy. Generally well-tolerated, but contraindicated in those with renal impairment, limiting its use in some elderly patients [72,73].

In the final analysis, the optimal medication hinges on a patient's unique presentation and necessitates collaborative decision-making with a healthcare provider. By meticulously weighing the advantages and disadvantages of each treatment modality, healthcare professionals can craft an individualized therapeutic strategy to bolster long-term recovery for elderly patients with AUD.

Medication-Assisted Treatment for Nicotine-Use Disorder

Similar to OUD and AUD, selecting a medication for NUD entails a comprehension of the pros and cons of each medication. This comparative analysis (Table 3), highlights features of MAT for NUD: NRT, bupropion, and varenicline.

**Table 3:** Comparative analysis of medication-assisted treatment for nicotine-use disorder NUD: nicotine-use disorder, NRT: nicotine replacement therapy

Aspect	NRT	Bupropion	Varenicline
Mechanism of Action	Provides controlled doses of nicotine to alleviate withdrawal symptoms and cravings, helping smokers quit by replacing nicotine obtained from smoking with a less harmful alternative [75].	Inhibits the reuptake of norepinephrine and dopamine, which helps reduce cravings and withdrawal symptoms, while also addressing depressive symptoms associated with NUD [78].	Partial agonist at nicotinic acetylcholine receptors (nAChR), reducing cravings and withdrawal symptoms while blunting the rewarding effects of smoking [81].
Effectiveness in Reducing Cravings	Increases the rate of smoking cessation by 50-70% when used as monotherapy. Effectiveness is further enhanced when combined with behavioral therapy [75,77]. Particularly effective in elderly smokers, with increased cessation success noted [76].	Doubles the odds of quitting smoking compared to placebo. Effective in increasing quit rates and continuous abstinence, particularly in elderly patients [78,80]. Also beneficial for patients with comorbid conditions such as cardiovascular disease [78].	Highly effective in promoting smoking cessation, particularly when combined with behavioral counseling. Some studies suggest it is more effective than NRT, although effectiveness in older adults varies [82,83]. Shows long-term effectiveness, especially when used in adaptive treatment approaches [84,86,87].
Safety and Tolerability	Generally safe and well-tolerated. Common side effects include localized skin irritation from patches and occasional gastrointestinal discomfort. Rare cases of chest pain and palpitations have been reported but are infrequent [75].	Well-tolerated with a favorable safety profile, even in the presence of comorbidities. Common side effects include insomnia and dry mouth, which are generally mild and manageable [80].	Generally well-tolerated, with the most common side effects being gastrointestinal disturbances. Previous concerns about neuropsychiatric and cardiovascular risks have been largely mitigated, but caution is still recommended, especially in older populations [85].
Accessibility	Widely accessible with various delivery methods, allowing flexibility in treatment. Available over-the-counter in many forms, making it convenient for self-management [75].	Prescription-only medication but considered cost-effective compared to NRT. Accessibility is good, especially when prescribed as part of a smoking cessation program [79].	Prescription-only, but increasingly used in clinical settings for smoking cessation. Adaptive treatment approaches and early initiation can improve accessibility and effectiveness in older adults [86,87].
Impact on Treatment Engagement	High engagement due to the availability of multiple forms and the ability to cater to individual preferences. Combining NRT with behavioral therapy further enhances engagement and success rates [75,77].	Bupropion's effectiveness in treating both NUD and comorbid depressive symptoms can improve engagement, particularly in older adults. The sustained-release formulation also supports better adherence [78,80].	High engagement when used as part of an adaptive treatment approach. Combining varenicline with behavioral counseling has been shown to enhance long-term engagement and success in smoking cessation [86,87].
Impact on Co-morbidities	Safe for use in elderly patients and those with comorbid conditions. The variety of delivery methods allows for customization based on patient needs and preferences [75].	Effective in treating NUD in patients with comorbid conditions such as cardiovascular disease. Also addresses depressive symptoms, making it suitable for patients with co-occurring mental health conditions [78,80].	Suitable for use in older adults, with previous safety concerns largely addressed. Effective in long-term smoking cessation, particularly when tailored to individual patient needs and combined with non-pharmacological interventions [83,86].
Overall Suitability for Older Adults	Highly suitable due to its safety, tolerability, and flexibility in dosing. Effective in promoting smoking cessation in elderly smokers, particularly when combined with behavioral therapy [75,77].	Well-suited for older adults due to its effectiveness in treating both NUD and co-occurring depressive symptoms. Its safety profile and potential cost-effectiveness make it a strong option for elderly patients [78,80].	Viable option for smoking cessation in older adults, especially with adaptive treatment approaches. Its targeted action and long-term effectiveness make it a compelling choice, though individual tolerance and potential side effects should be monitored [83,86,87].

To conclude, NRT offers flexibility and accessibility through various delivery methods, making it a versatile option for elderly smokers. Bupropion, with its dual mechanism of action and favorable safety profile, is especially effective for those with co-occurring depressive symptoms and comorbid conditions. Varenicline, while highly effective in promoting long-term cessation, benefits from adaptive treatment approaches and remains a strong option for older adults, particularly when combined with behavioral counseling. Each medication has distinct advantages, and the choice of therapy should be individualized based on the patient's health status, preferences, and potential barriers to treatment adherence.

Non-pharmacological interventions

These interventions strive to comprehensively target the psychological, social, and behavioral underpinnings of addiction. They encompass CBT, motivational interviewing, contingency management, group therapy, support groups, and family therapy.

Cognitive Behavioral Therapy 

CBT is key in addressing SUDs in older adults. It targets unhealthy thought patterns, behaviors, and emotional responses associated with substance use. By employing techniques like cognitive restructuring (identifying and challenging distorted thinking), behavioral activation (increasing healthy activities), coping skills training (developing strategies to manage cravings), and relapse prevention planning, CBT empowers older adults to break free from the cycle of addiction [88]. Research provides compelling evidence for the efficacy of CBT in this population [44]. A study by Hu et al., demonstrated the effectiveness of CBT in older adults especially when combined with pharmacotherapy, with this combination enhancing treatment outcomes more than when only pharmacotherapy is utilized [89]. This study also revealed the effectiveness of CBT when combined with taper, which improved the patients symptoms over the initial three-month period [89]. In AUD however, the Lin et al. study, showed that the twelve-step facilitation is more effective than CBT [4]. The Evans study showed that CBT when meticulously tailored to address the cognitive and emotional needs of elderly patients, can significantly reduce substance use and strengthen their coping abilities [90]. The study also underscored the importance of several modifications to accommodate changes with aging like age-related cognitive decline and potential sensory impairments (an example being hearing loss, which 50-75% of older adults above 70 years have) [90]. These crucial modifications encompass several key aspects: (1) Pace and Structure. Slower-paced sessions with a more structured format can benefit elderly patients who may have slower processing speeds or cognitive limitations [38,90]. (2) Age-Specific Focus. Studies showed that addressing issues specific to aging, such as coping with loss, managing chronic pain, and navigating social isolation, enhances the relevance and effectiveness of CBT for older adults [38,90]. (3) Family and Caregiver Involvement. Studies also reveal that integrating family members and caregivers into the therapy session fosters additional support and reinforces positive behavioral changes for the patient [38,90]. (4) Technology Integration. A study by Nesvag and Mckay revealed that utilizing teletherapy platforms or computer-based CBT programs can improve accessibility for elderly patients facing mobility limitations or residing in remote areas [91]. These tools serve as valuable complements to in-person therapy, providing additional resources for practice and reinforcement of learned skills.

In conclusion, CBT stands out as a highly effective non-pharmacological treatment for SUDs in older adults. By simultaneously targeting both the cognitive and behavioral aspects of substance use, CBT empowers elderly patients to develop healthier coping mechanisms, reduce substance dependence, and ultimately improve their quality of life.

Motivational Interviewing 

MI is a non-pharmacologic intervention that aligns seamlessly with the unique needs of elderly patients with SUDs [38,92]. The core strength of MI lies in its emphasis on empathy and respect for patient autonomy. Unlike traditional models that might feel confrontational or judgmental, MI fosters a safe and supportive environment, with therapists employing MI techniques actively listening to the patients, and acknowledging the difficulties they face [38,92]. The effectiveness of MI in this demographic has been demonstrated in several studies. An example is the study by Cummings et al., which identified several cases in the general patient population receiving MI [93]. By employing MI strategies, a patient was reported to maintain sobriety for four months, having identified fear of delirium tremens recurrence and her longing to be with her grandchildren as strong motivational factors [93]. The study concluded that MI may help mitigate harm and risk and maintain the quality of life of older adults [93]. The study by Satre et al. also affirmed the effectiveness of MI in patients with AUD and depression, which at six months was revealed to be more effective than control in reducing the rate of hazardous drinking (four or more drinks in a day for women, five or more drinks in a day for men; p = .060) [94]. On the contrary, a study by Schmidt et al., reported that the fidelity of the MI intervention delivered to participants did not lead to better treatment outcomes [95]. This study also revealed that MI may have reduced effectiveness in populations already motivated to change [95]. Comparing MI to non-directive listening and self-change condition in older adults with AUD, as carried out in a study by Kuerbis et al., revealed that MI outperformed non-directive listening but not self-change condition [96].

While MI can be most beneficial when used in conjunction with other evidence-based treatments, such as CBT [92], and offers a promising approach for treating SUDs in older adults, further research is needed to determine its specific effectiveness in this demographic conclusively.

Contingency Management

Contingency Management (CM) is a behavioral technique based on operant conditioning principles that utilizes the power of positive reinforcement to promote recovery and functions through a two-pronged approach: a system of increasing rewards and consistent monitoring [38]. Several studies have demonstrated the effectiveness of CM, an example being the Cahill et al. study which revealed the incentives significantly increased cessation rates [97]. The study showed an OR of 1.42 (95% CI 1.19 to 1.69; 17 trials, 20 comparisons, 7715 participants) for quitting smoking with incentives at the longest follow-up (six months or more) compared with controls [97]. Similarly, the Petry et al. study revealed that CM is effective for a wide range of substance use disorders (including stimulants, opioids, marijuana, nicotine, etc), can be integrated with nearly any form of psychotherapy or pharmacotherapy and remains effective regardless of patients’ characteristics (such as age, sex, race, etc) or pre-existing conditions [98]. Regarding its integration with other forms of psychotherapy, the combination of CM with group therapy is beneficial, resulting in positive outcomes [99]. The Petry et al. study also revealed that patients receiving CM for SUD achieved an average of 4.4 weeks of abstinence compared to 2.6 weeks for patients receiving standard care alone [98]. This study, however, described CM as the least implemented empirically-based intervention in clinical settings and attributed this phenomenon to concerns like harmony with other treatment modalities, motivation for change, durability, and economics [98]. As regards the harmony with other treatment modalities, studies revealed improved outcomes when CM is combined with other interventions [98]. The concern of motivation has also been refuted by recent studies revealing that CM does not adversely affect the motivation to change behaviors related to substance misuse [98]. The issues of durability and economics are still being debated, as it has been revealed that there are no or possibly few long-term benefits, as well as reimbursements after incentives have been given to the patients [98]. To sum up, CM remains a useful approach for enhancing abstinence and treatment adherence in older adults with SUDs.

Group Therapy and Support Groups

Group therapy and support groups offer a multifaceted approach to addressing SUD in older adults. These interventions provide crucial social support, alleviate the burden of isolation, and foster a sense of shared purpose through collective experiences [38]. The effectiveness of group therapy and support groups has been extensively demonstrated in studies. An example is the study by Cooper et al., which revealed the effectiveness of support groups in improving clinical outcomes, self-efficacy, and recovery [47]. Similarly, a study by Lopez et al. revealed that performing CBT in groups, as well as CM is more effective in reducing substance use [100]. This study also underscored the effectiveness of support groups in reducing substance use and demonstrated that combining group therapy with MAT was more effective in reducing opioid use than MAT alone [100]. Studies have also demonstrated that group therapy, like motivationally enhanced group counseling, can improve participation in formal treatment programs as well as 12-step groups, especially in severe cases [101]. On the contrary, some studies downplay the effectiveness of group therapy. One example is the study by Lo Coco et al. which revealed that the effectiveness of group therapy on substance use frequency and the symptoms of SUDs were not significant [102]. This study also revealed that there were no differences in the abstinence rates between group therapy and control groups [102]. However, Lo Coco et al. pointed out the significant positive effect group therapy had on mental state when compared to no treatment [102]. Another example is the Weiss et al. study which revealed that there were no differences in the treatment outcomes between group and individual therapies [103].

In conclusion, by tailoring group therapy and support groups to the unique needs of older adults with SUDs, these interventions can enhance the recovery process.

Family Therapy

Family therapy (FT) represents a transformative approach that recognizes the profound influence family dynamics have on addiction and recovery [38]. Studies have shown that incorporating family therapy into the treatment plan of patients with SUDs results in positive outcomes. For instance, the study by Hu et al. underscores the importance of including family members in the management of SUDs depending on the level of patient autonomy and severity of the SUD [89]. The Evans study demonstrated that integrating family therapy and CBT enhanced patient outcomes [90]. Another study by Hogue et al. provided an evidence-based update on the effects of FT spanning a decade [104]. While highlighting the various types of FTs (like systemic FT, behavioral FT, etc.), this study demonstrated the effectiveness of FT in both scenarios of exclusive treatment and as part of a multicomponent treatment, adding that age did not influence the degree of benefit from these therapies [104]. Furthermore, a study by Rowe affirmed that family-based treatment models are increasingly recognized as among the most effective approaches for managing drug abuse across all age groups, proving to be a viable alternative to traditional methods [105].

Ultimately, family therapy empowers families of elderly SUD patients to overcome challenges, build support, and create a recovery-friendly environment through collaboration and tailored interventions.

Comparative analysis of non-pharmacological interventions

Table 4 summarizes the comparative analysis of non-pharmacological interventions.

**Table 4:** Comparative analysis of non-pharmacological interventions

Therapeutic Approach	Key Features	Efficacy	Considerations for Older Adults
Cognitive Behavioral Therapy (CBT)	Focuses on altering unhealthy thoughts and behaviors. Techniques include cognitive restructuring, behavioral activation, coping skills training, and relapse prevention [88].	Highly effective, especially when combined with pharmacotherapy [44,89]. Less effective in Alcohol Use Disorder (AUD) compared to Twelve-Step Facilitation [4]. Tailoring CBT for cognitive and emotional needs improves outcomes [90].	Requires modifications like slower pacing, structured sessions, and addressing age-specific issues (e.g., loss, chronic pain) [38,90]. Involving family/caregivers and using teletherapy can enhance accessibility and support [38,90,91].
Motivational Interviewing (MI)	Emphasizes empathy and patient autonomy, creating a supportive environment [38,92].	Effective in reducing hazardous drinking and maintaining sobriety, though outcomes vary [93,94]. Potentially less effective in already motivated individuals [95].	Often used in conjunction with other therapies like CBT for better outcomes [92]. More research needed on specific effectiveness in older adults [96].
Contingency Management (CM)	Uses positive reinforcement and consistent monitoring to promote recovery [38].	Increases cessation rates for various substances, including nicotine and opioids [97,98]. Improved outcomes when combined with other therapies [99].	Concerns about long-term benefits and economic feasibility [98]. Effective for enhancing abstinence and treatment adherence [98].
Group Therapy and Support Groups	Provides social support, reduces isolation, and fosters shared experiences [38].	Effective in improving self-efficacy and recovery when combined with other interventions [47,100].	Mixed results on substance use outcomes; more effective in improving mental state [102]. Tailoring to specific needs of older adults is essential [102].
Family Therapy (FT)	Involves family in the recovery process, addressing family dynamics influencing addiction [38].	Recognized as one of the most effective approaches across all age groups [104,105]. Enhances patient outcomes, especially when combined with other therapies like CBT [90].	Family involvement is crucial, especially in scenarios with severe SUDs or reduced patient autonomy [89]. Tailored to address specific family dynamics and age-related challenges [38,104].

To sum up, each therapy has unique strengths: CBT is particularly effective when tailored to the cognitive and emotional needs of older adults. MI enhances motivation but may have a limited impact on those already motivated. CM effectively promotes abstinence, though concerns about cost and long-term effectiveness remain. Group Therapy and Support Groups offer crucial social support, especially when integrated with other therapies, while FT uses family involvement to aid recovery across all ages. Integrating these therapies and focusing on the specific needs of older adults can greatly enhance their recovery outcomes and overall well-being.

Holistic care models

Integrated care models present a robust paradigm for the management of SUDs among the elderly. These models transcend traditional, siloed approaches by seamlessly amalgamating medical, psychological, and social services. They address cognitive, physical, mental, financial emotional as well spiritual issues that hinder treatment and improve recovery [38]. Key components of holistic care models are multidisciplinary teams, centralized service access, care coordination, and patient-centered care [38]. Studies have demonstrated the effectiveness of this approach extensively. The Lin et al. study revealed that this approach is highly effective in catering to comorbidities common in older adults [4]. Similarly, the Kelly et al. study revealed that the combination of psychotherapy and pharmacotherapy is the most effective approach for patients with comorbidities [106]. The study by Savic et al. revealed that integrated care improved the mental health outcomes of patients with SUDs and that patients reported better satisfaction rates with this approach than with standard treatment [13]. This study also revealed that as regards integrated care, at no additional cost, abstinence rates were higher and there was an improvement in medical well-being and functioning outcomes up to nine years post-treatment entry [13]. Additionally, another study by Tai and Volkow supported the effectiveness of these models by revealing that patients who had both SUDs and other physical conditions utilized inpatient care emergency room visits more when they were assigned to a non-integrated treatment group compared to those who were assigned to an integrated management group [107]. Regarding the multidisciplinary teams in holistic care, the Townley and Dorr study underscored the importance of primary care physicians (who act as the first point of contact) in the effectiveness of integrative approaches [108].

Holistic models are not only important for the treatment of SUDs but are as vital in preventing SUDs as demonstrated in a study by Schainker et al. [109]. This study revealed that by integrating strategies like psychoeducation for pain management, biblio-prevention, e-health for pain education and management, group therapy, and individual interventions, SUDs can be prevented, especially in those individuals and populations considered at-risk [109]. Further in this study, it was revealed that multidisciplinary strategies were more effective than the usual care for pain management [109].

Overall, holistic care models, by combining medical, psychological, and social services, offer a comprehensive framework for tackling the complex challenges faced by elderly patients with SUD.

Policy recommendations

Funding and Resource Allocation

Boost investment in holistic care models by allocating funds to develop and implement these models. Also, give grants and financial incentives to facilitate the formation and maintenance of multidisciplinary healthcare teams.

Enhancing Workforce Capacity

Mandate specialized training programs for healthcare providers to equip them with the knowledge and skills necessary to address the unique complexities of this population. Offer continuing education credits to incentivize ongoing professional development and ensure that practitioners remain at the forefront of evidence-based practices in the field of geriatric SUD treatment.

Accessibility and Infrastructure

Create centralized service hubs to provide elderly patients with a single location to access comprehensive medical, psychological, and social services, effectively minimizing barriers to care. Furthermore, expand telehealth services through supportive policies, increased funding for telehealth infrastructure, and provider training.

Comprehensive Case Management and Care Integration

Fund case management services to optimize care for elderly patients with SUDs. Mandate integrated electronic health records to facilitate efficient communication and information sharing among healthcare providers.

Personalized Care and Family Engagement

Strengthen support networks by funding family therapy programs. Prioritize patient autonomy by promoting patient-centered care practices, ensuring that patients are active partners in their treatment journey.

Research and Data Collection

Prioritize research funding to enhance understanding of SUDs among the elderly, informing the development of targeted interventions. Establish a national database to track prevalence, treatment outcomes, and long-term effects, facilitating evidence-based policymaking and resource allocation.

Community Education and Prevention Initiatives

Implement targeted public health campaigns to raise awareness about SUD risks in the elderly population, reduce stigma, and encourage early intervention. Develop prevention programs specifically addressing chronic pain management, mental health support, and social isolation to mitigate SUD risk factors in older adults.

Health Coverage and Reimbursement Policies

Mandate comprehensive insurance coverage for integrated care services, including medical, psychological, and social support, for elderly patients with SUDs. Implement parity in reimbursement for telehealth services to promote access to care for this vulnerable population.

These policy recommendations offer a roadmap for transforming the care landscape for older adults with SUDs. By implementing these strategies, governments and healthcare organizations can create a more supportive and effective system of care.

## Conclusions

In conclusion, effectively managing SUD in the elderly population necessitates a comprehensive treatment strategy. The contemporary landscape of SUD treatment underscores the criticality of individualized interventions that acknowledge the unique physiological, psychological, and social challenges confronting older adults. The assessment of therapeutic interventions for this demographic has yielded valuable insights into their relative strengths and limitations. However, the most promising course of action lies in an integrated, holistic approach. This approach fosters a synergy between medical, psychological, and social services, thereby ensuring a well-coordinated and continuous treatment plan. By leveraging the expertise of a diverse team of healthcare professionals, centralized access to services, and a patient-centered care philosophy, this comprehensive framework addresses the multifaceted nature of SUD in elderly patients. This strategy not only mitigates the risk of relapse but also demonstrably improves the overall quality of life for older adults battling addiction. Indeed, the future of geriatric SUD management hinges upon recognizing and addressing the unique needs of this vulnerable population, ensuring they receive the comprehensive care and support necessary to navigate their path toward recovery.
